# Antibiotics-induced depletion of mice microbiota induces changes in host serotonin biosynthesis and intestinal motility

**DOI:** 10.1186/s12967-016-1105-4

**Published:** 2017-01-13

**Authors:** Xiaolong Ge, Chao Ding, Wei Zhao, Lizhi Xu, Hongliang Tian, Jianfeng Gong, Minsheng Zhu, Jieshou Li, Ning Li

**Affiliations:** 1 Department of General Surgery, Jinling Hospital, Medical School of Nanjing University, 305 East Zhongshan Road, 210002 Nanjing, China; 2 Model Animal Research Center and MOE Key Laboratory of Model Animal for Disease Study, Nanjing University, Nanjing, China; 3 Department of Medical Genetics, and Jiangsu Key Laboratory of Molecular Medicine, Medical School of Nanjing University, Nanjing, China

**Keywords:** Intestinal microbiota, Antibiotics, Gut motility, Serotonin, Bile acids

## Abstract

**Background:**

The gastrointestinal motility is affected by gut microbiota and the relationship between them has become a hot topic. However, mechanisms of microbiota in regulating motility have not been well defined. We thus investigated the effect of microbiota depletion by antibiotics on gastrointestinal motility, colonic serotonin levels, and bile acids metabolism.

**Methods:**

After 4 weeks with antibiotics treatments, gastrointestinal and colon transit, defecation frequency, water content, and other fecal parameters were measured and analyzed in both wild-type and antibiotics-treated mice, respectively. Contractility of smooth muscle, serotonin levels, and bile acids levels in wild-type and antibiotics-treated mice were also analyzed.

**Results:**

After antibiotics treatment, the richness and diversity of intestinal microbiota decreased significantly, and the fecal of mice had less output (*P* < 0.01), more water content (*P* < 0.01), and longer pellet length (*P* < 0.01). Antibiotics treatment in mice also resulted in delayed gastrointestinal and colonic motility (*P* < 0.05), and inhibition of phasic contractions of longitudinal muscle from isolated proximal colon (*P* < 0.01). In antibiotics-treated mice, serotonin, tryptophan hydroxylase 1, and secondary bile acids levels were decreased.

**Conclusion:**

Gut microbiota play an important role in the regulation of intestinal bile acids and serotonin metabolism, which could probably contribute to the association between gut microbiota and gastrointestinal motility as intermediates.

## Background

The human gastrointestinal tract is a diverse microbial community which is composed of hundreds of microbial species [1]. Studies have focused on the role of gut microbiota on host physiology, which show the microbiota affect gut motility, tissue regeneration (in particular of the villi), gut-associated lymphoid tissue (GALT) maturation, and intestinal barrier function [2]. Accumulating evidence also suggest that gut microbiome could play a pivotal role in the immune system development, protecting host against invading pathogens, and nutrient reclamation and absorption, thus microbiome might avert carcinogenesis, like in breast cancer [3]. Alterations in the composition of gut microbiota may lead to dysfunction of motility, which might be one of the pathogenic factors in slow transit constipation (STC) [4, 5].

The germ-free animals are used in many studies to show the influence of gut microflora on gut motor function. The cecum of germ-free rats was reported to be enlarged up to 10 times its normal size, and both gastric emptying and intestinal transit were prolonged compared to conventionally raised animals [6]. Barbara et al. [6] suggested that gut microflora affected intestinal motility mainly by bacterial substances or end products of bacterial fermentation, intestinal neuroendocrine factors, or mediators released by the gut immune system.

The monoamine serotonin (5-hydroxytryptamine [5-HT]) is an important gut neuroendocrine factor which is synthesized mainly by enterochromaffin cells (ECs), and has been demonstrated to regulate gut motility in certain pathways. Furthermore, almost 90% of serotonin is synthesized in colonic ECs [7]. Tryptophan hydroxylase 1 (TPH1) and TPH2 are rate limiting enzymes in mucosal and neuronal serotonin biosynthesis, respectively [8]. It is suggested that fecal pellets contributed to the release of 5-HT from ECs through activating local mucosal reflexes and stretch reflexes of colonic migrating motor complex (CMMC) to facilitate propulsion [9]. Fukumoto et al. [10] reported that 5-HT from ECs could stimulate 5-HT_3_ receptors which were located on the vagal sensory fibers, and the sensory information was transferred to the vagal efferent or it stimulated the release of acetylcholine which affected muscle contraction. 5-HT could also cause the release of Ca^2+^ from the sarcoplasmic reticulum after it activated 5-HT_3_ receptors and the inositol 1,4,5-trisphosphate pathway, which induced contraction of colonic myocytes [11]. Yano et al. [12] suggested that gut microbes regulated the 5-HT level in colon, which impacted intestinal motility and hemostasis. Bile acids (BAs) in intestine were reported to exert opposite functions, which inhibited motility in the small intestine and stimulated motility in the large intestine [13]. Patients with cholestatic disease had a decreased colonic delivery of free BAs, and they would suffer from constipation [14]. It is reported that a G protein-coupled receptor TGR5 could be activated by BAs [15]. Intestinal BAs could promote peristaltic contractions in the colon via TGR5 with the release of 5-HT and calcitonin gene-related peptide (CGRP) [13]. But few systematical studies focus on the relationship among BAs, 5-HT, microbiota, and gut motility.

Germ-free mice model has been used in several studies which were interested in the relationship between gut microbiota and motility. To the best of our knowledge, the germ-free mice model has several limitations as germ-free mice are born in aseptic conditions, which may not only affect the development of the gut motility system, but also the immunity system and metabolic function [16]. In some studies, broad-spectrum antibiotics were used to deplete gut commensal microflora for creating pseudo-germ-free mice [17–20]. The antibiotics-treated method has been used to prove the reversibility of microbial effects on host 5-HT and gut motility [12].

The present study was designed to evaluate whether depletion of gut microbiota by antibiotics could influence gut motility and whether this effect was mediated by regulating host serotonin metabolism.

## Methods

### Animals and antibiotics treatment protocol

All procedures were conducted according to Model Animal Research Center and were approved by the Experimentation Ethics Review Committee of Nanjing University. Twenty male C57BL/6 mice (6–7 weeks old) were obtained from Model Animal Research Center at Nanjing University and adapted to environmental conditions for one week before experiments. Mice were housed in a temperature and humidity-controlled room with a 12 h light/dark cycle (19.0 ± 1 °C, 55% humidity, and fed on a maintenance diet). For the induction of gut dysbiosis, ten mice were treated with broad-spectrum antibiotics in drinking water for 4 weeks (ampicillin, 1 g/L, Sigma; neomycin sulfate, 1 g/L, Sigma; metronidazole, 1 g/L, Sigma; vancomycin, 500 mg/L, Sigma) [18].

### Analysis of gut microbiota

After antibiotics, stool samples of mice were collected in a sterile micro-tube, which were frozen immediately in liquid nitrogen and stored in a −80 °C freezer until analysis. Total DNA was isolated using a Wizard Genomic DNA Purification Kit, which was following the manufacturer’s instructions (Promega, Madison, USA). The relative abundance of bacteria was measured using 16S rRNA analysis, which was performed at the laboratory of BGI (Huada Gene Institute). Primers were designed to amplify the V3–V4 hypervariable region according to the manufacturer’s recommendations [21]. Then bioinformatics analysis was performed as described [22]. The mother v.1.27.0 Standard Operation Procedure (SOP) was used to analyze high quality sequence reads [23].

### Total gastrointestinal transit

After fasted overnight with free access to water, mice were administrated by gavage with a semiliquid solution (0.1 mL) containing Evans blue (5%) and methyl cellulose (1.5%) (M0262, Sigma) [13]. Then fecal pellets were monitored at 10 min intervals for the presence of the first blue pellet, and the time for expulsion of the first blue pellet was measured [24].

### Measuring colonic transit

After fasted overnight with free access to water, the colonic transit of mice was measured with a bead expulsion test. A 3-mm glass bead was inserted into the colon (2 cm proximal to the anal) using a plastic Pasteur pipette lightly lubricated with lubricating jelly as described [25]. The time until bead expulsion was measured.

### Fecal parameter measurements

Freely feeding mice were observed for 8 h, and the number of pellets was counted every 2 h. Fecal water content was measured by comparing the weight of the pellets at the end of the experiment and after drying (24 h at 60 °C). Finally, fecal dimensions of each mice were measured.

### Macroscopic assessment

Animals were weighted every week from the beginning to the end of experiments. Mice were euthanized by cervical dislocation, and the whole intestine (from stomach to anus) were excised. The length of the whole intestine and colon was measured. The weight of cecum with their contents was also measured.

### Analysis of smooth muscle contractility

Strips of longitudinal (6 mm in length) muscle from proximal colon were mounted in a 37 °C organ bath with a force transducer (MLT0202; AD Instruments, Spain) connected to a PowerLab (AD Instruments, Australia) recording device. Briefly, Ca^2+^-free HEPES-Tyrode (H-T) buffer (140.6 mmol/L NaCl, 2.7 mmol/L KCl, 1.0 mmol/L MgCl_2_, 10 mmol/L HEPES, and 5.6 mmol/L glucose, pH 7.4) was used to wash out the lumen contents of proximal colon segments. Then, segments were transferred to the organ bath and equilibrated in H-T buffer (137.0 mmol/L NaCl, 2.7 mmol/L KCl, 1.0 mmol/L MgCl_2_, 1.8 mmol/L CaCl_2_, 10 mmol/L HEPES, and 5.6 mmol/L glucose, pH 7.4) for 15 min. The resting tension was set to 0.5 g prior to force measurement. The isotonic contractions of longitudinal muscle was recorded for 1 h, with H-T buffer being changed every 15 min. The mean amplitude of the basal tension and the frequency of phasic contractions were measured for 30 min after experiments. The detailed force assays were performed according to our previously described methods [26, 27].

### Serotonin measurements

ELISA was used to assess the serotonin in supernatant of tissue homogenates according to the manufacturer’s instructions (DEE5900, Germany). Readings from tissue samples were normalized to total protein content as detected by BCA assay as described [12].

### RNA preparation and qRT-PCR

The entire length of the mouse colon were washed in H-T buffer, flushed with H-T buffer to remove luminal contents. Then the total RNA was extracted from 1 cm regions of proximal mouse colon using TRIzol reagent (Invitrogen). The qRT-PCR assay was performed by a ABI Prism 7100 Sequence Detection System and 96-well optical plates (Applied Biosystems). The PCR primer sequences were as follows, M-TPH1: forward, 5′TCA AGC CCT TTG ATC CCA AG-3′; reverse, 5′-GAC ATC AAG GTC ATA CCG CAA C-3′.

### Immunofluorescence

Whole frozen sections were processed for indirect immunofluorescence and confocal microscopy as described [12], which were prepared from paraformaldehyde-fixed tissues. Briefly, 10-μm frozen sections of proximal colon samples were collected on coated slides. Then it was washed three times with PBS and blocked with 5% bovine serum albumin (sigma Aldrich) at room temperature for 30 min. After staining using the primary antibodies, rabbit anti-mouse CgA (1:500; Abcam), rat anti-mouse 5-HT (1:50; Abcam), and secondary antibodies conjugated to Alexa fluor 488 or 594 (Molecular Probes). Images were examined using a confocal microscope (Olympus, Germany).

### Cecal bile acids determination

Cecal contents from mice were collected, freeze-dried, and pulverized. BAs were analyzed using the LC–MS–MS (Liquid Chromatography–Mass Spectrometry and Liquid Chromatography–Tandem Mass Spectrometry). Each bile acid was identified by its relative retention time compared with that of standard BAs.

### Statistical analysis

Statistical analysis was performed using SPSS for windows version 19. Data were measured for normal distribution and plotted in the figures as mean ± SEM. The significance of the differences between groups was determined with Student t test (two comparisons) or one-way ANOVA (multiple comparisons). *P* < 0.05 was considered significant.

## Results

### Composition of intestinal microbiome in mice after antibiotics

**Fig. 1:**
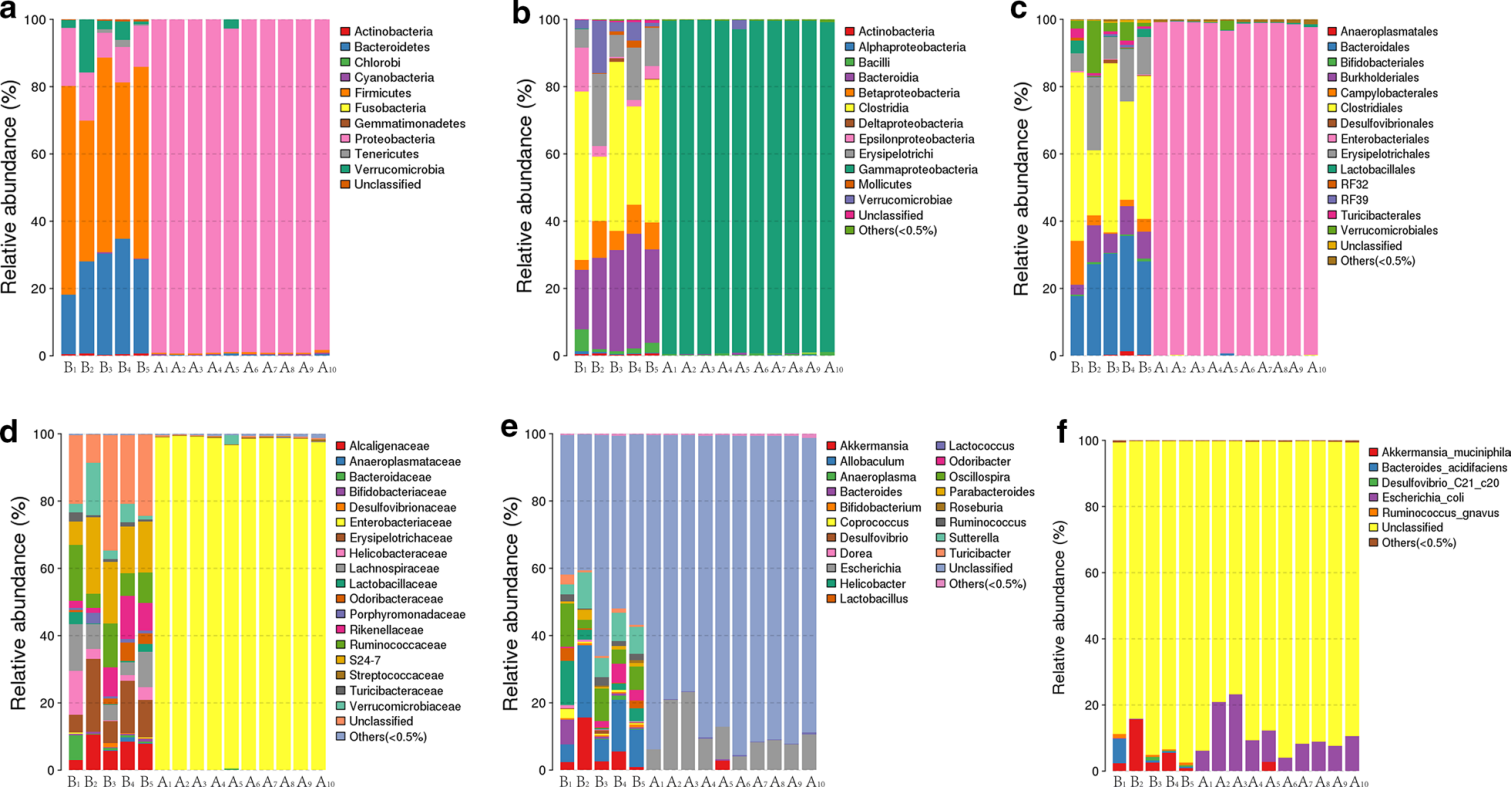
Composition of the mice intestinal microbiota after antibiotics at every level. And the relative abundance of microbiota at every level is measured and significant differences are found between two groups. **a** Phylum level; **b** class level; **c** order level; **d** family level; **e** geneus level; **f** species level. *B1*–*B5* SPF mice without antibiotics; *A1*–*A10* SPF mice with antibiotics

To determine the importance of commensal microbiota in gut motility, mice were subjected to a 4-week oral administration of antibiotics combination (ampicillin, neomycin sulfate, metronidazole, and vancomycin) [17, 18, 20, 28, 29]. The antibiotics resulted in changes in the composition of commensal bacteria examined by 16S rRNA analysis. After 4 weeks with antibiotics treatments, the average number of operational taxonomic unit (OTU) of mice decreased significantly from 383.4 ± 23.4 to 74.9 ± 3.1 (P < 0.01). Taxonomically, after antibiotics treatments, the abundance of intestinal microbiota decreased significantly in all levels as shown in Fig. 1(a–f) (Phylum, Class, Order, Family, Genus, and Species). At phylum level, only Proteobacteria accounted for more than 0.5% of all the microbiota in mice treated with antibiotics. On the contrary, in SPF mice without antibiotics, Actinobacteria (0.6%), Bacteroidetes (27.4%), Firmicutes (53.0%), Proteobacteria (12.3%), Tenericutes (0.8%), and Verrucomicrobia (5.4%) accounted for more than 0.5%. In addition, the species richness (Chao) decreased from 354.3 ± 42.9 to 98.3 ± 15.1 (P < 0.01), and α diversity (Shannon) decreased from 4.0 ± 0.4 to 0.5 ± 0.1 (P < 0.01).

### Depletion of gut microbiota affects fecal parameter and weight of cecum

**Fig. 2:**
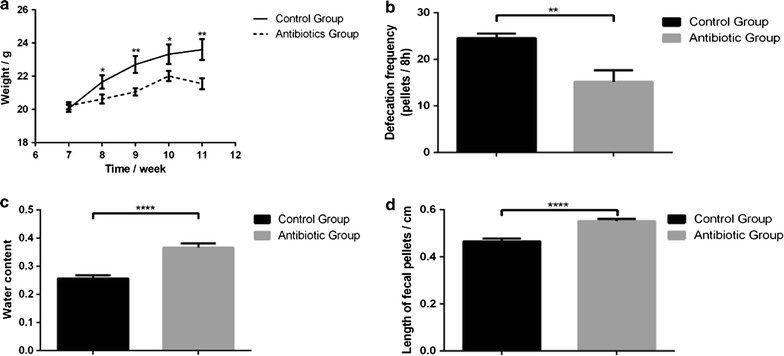
Effects of microbiota depletion by antibiotics on mice body weight (**a**), defecation frequency (**b**), fecal water content (**c**), and length of fecal pellets (**d**). Data are presented as mean ± SEM. *P < 0.05, **P < 0.01

**Fig. 3:**
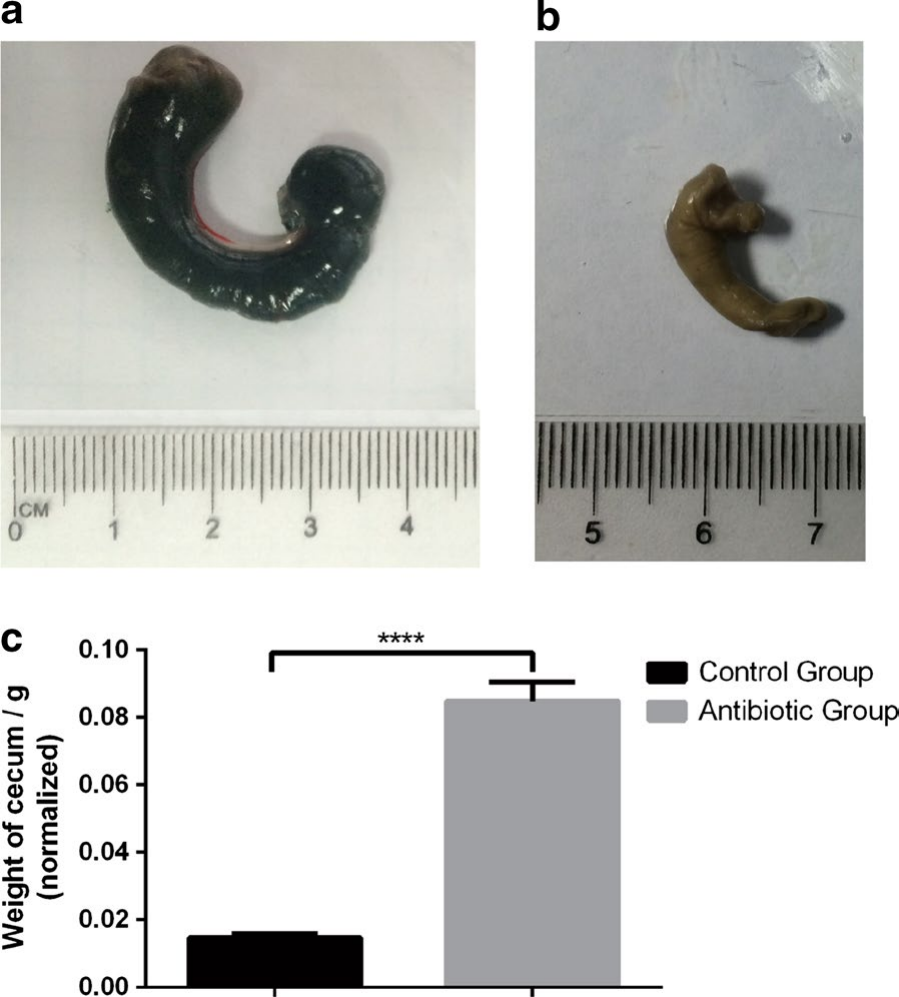
Effect of antibiotics on size and weight of cecum. **a** The cecum of antibiotics-treated group, **b** The cecum of control group, **c** The weight of cecum in antibiotics-treated and control groups after it was adjusted by weight of mice. Data are presented as mean ± SEM. ****P < 0.01

After 4 weeks with antibiotics treatments, mice had a significant tendency toward decreased in weight when compared to control every week (Fig. 2a). Antibiotics-treated mice had lower pellet frequency (24.5 ± 1.0/8 vs 15.2 ± 2.4/8 h, *P* < 0.05) (Fig. 2b), higher water content (25.6 ± 1.1 vs 36.7 ± 1.5%, *P* < 0.01) (Fig. 2c), and longer pellet size (0.47 ± 0.01 vs 0.55 ± 0.01 cm, *P* < 0.01) (Fig. 2d). The cecum of antibiotics-treated mice was significantly larger than its normal size after it was adjusted by weight of mice (0.015 ± 0.001 vs 0.085 ± 0.006 g, *P* < 0.01) (Fig. 3).

### Antibiotics affect gastrointestinal and colonic transit

**Fig. 4:**
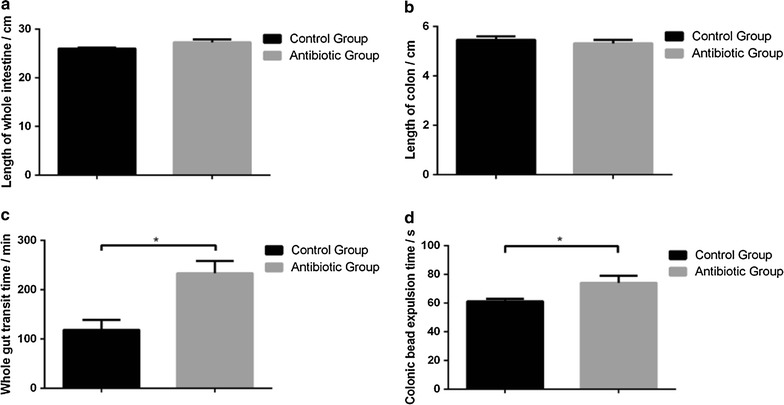
Antibiotics induce changes of length of whole intestine (**a**), length of colon (**b**), and gastrointestinal (**c**) and colonic transit (**d**). Data are presented as mean ± SEM. *P < 0.05

Evans blue test and bead expulsion test were used to evaluate gastrointestinal and colonic transit. Antibiotics-treated mice had the similar length of the whole gut and colon with the control (The whole intestine: 26.0 ± 0.2 vs 27.3 ± 0.6 cm, *P* = 0.06; Colon: 5.5 ± 0.1 vs 5.3 ± 0.1 cm, *P* = 0.52) (Fig. 4a, b). In Evans blue test, the transit time showed a slower intestinal transit in antibiotics-treated mice (118.0 ± 20.6 vs 233.3 ± 24.9 min, *P* < 0.05) (Fig. 4c). To specifically examine colonic transit, a bead expulsion test was used in this study [13, 30]. The time until bead expulsion in mice treated with antibiotics was slower than the control (61.0 ± 1.9 vs 74.0 ± 4.8 min, *P* < 0.05) (Fig. 4d).

### Gut microbiota regulate contractility of proximal colonic muscle

**Fig. 5:**
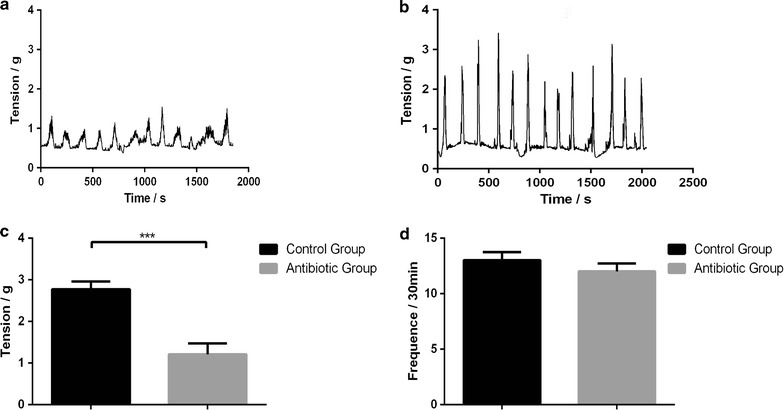
Gut microbiota depletion by antibiotics regulates colonic contractility. Recordings were made of spontaneous phasic contractions of proximal colon. Representative recordings from antibiotics-treated mice (**a**) and control mice (**b**). Tension (**c**) and frequency (**d**) normalized to basal values in antibiotics-treated and control mice. Data are presented as mean ± SEM. ***P < 0.01

As antibiotics-treated mice had a decreased fecal output and delayed intestinal transit, it raised the possibility that commensal microbiota might affect the spontaneous contraction of intestinal smooth muscle. After antibiotics treatments, it showed that the inhibition of spontaneous phasic contractions of colonic longitudinal muscle was found in antibiotics-treated mice. However, only the muscle tension was inhibited, the frequency of contractions was not changed (Tension: 2.77 ± 0.18 vs 1.21 ± 0.26 g, *P* < 0.01; Frequency: 13.0 ± 0.7/30 vs 12.0 ± 0.7/30 min, *P* = 0.36) (Fig. 5).

### Gut microbiota modulate metabolites and host colonic serotonin levels

**Fig. 6:**
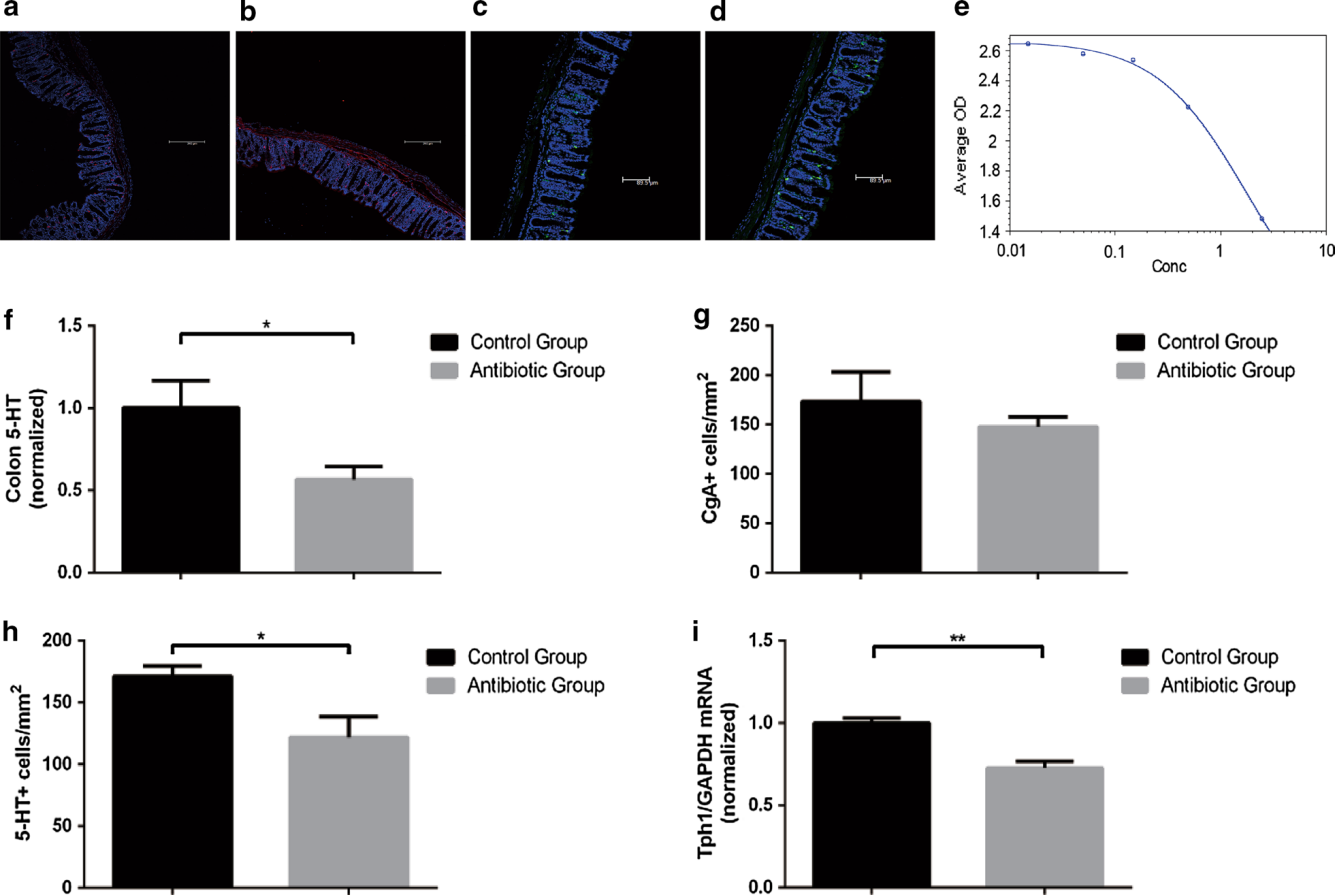
Serotonin metabolites are affected by antibiotics in mice. **a** Representative images of colons stained for chromogranin A (CgA) in SPF mice without antibiotics. **b** Representative images of colons stained for chromogranin A (CgA) in SPF mice with antibiotics. **c** Representative images of colons stained for 5-HT in SPF mice without antibiotics. **d** Representative images of colons stained for 5-HT in SPF mice with antibiotics. **e** Standard curve of 5-HT in colon by ELISA. **f** Levels of colon 5-HT relative to total protein. Data are normalized to colon 5-HT relative to total protein in control mice. **g** Quantitation of CgA^+^ cell number per area of colonic epithelial tissue. **h** Quantitation of 5-HT^+^ cell number per area of colonic epithelial tissue. **i** Colonic expression of TPH1 relative to GAPDH. Data are normalized to levels in control mice. Data are presented as mean ± SEM. *P < 0.05, **P < 0.01

**Fig. 7:**
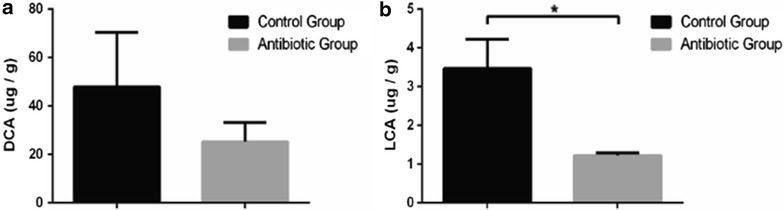
Effect of antibiotics on levels of secondary bile acids in different groups. **a** Deoxycholic acid (DCA) level in control group and antibiotic group. **b** Lithocholic acid (LCA) level in control group and antibiotic group. Data are presented as mean ± SEM. *P < 0.05

ELISA was used to measure the colonic 5-HT levels. The results showed that “microbiota depletion” by antibiotics combination resulted to a deficient colonic 5-HT in mice (Fig. 6f). Although there was no difference in the abundance of chromogranin A-positive (CgA^+^) ECs between antibiotics-treated mice and SPF mice, a difference in 5-HT^+^ cell was found (Fig. 6a–d, g, h). It showed that in antibiotics-treated mice, the expression of TPH1 was decreased (Fig. 6i). Moreover, intestinal bile acids were tested to find out the changes of metabolism in intestine with antibiotics treatments. Levels of lithocholic acid (LCA) in antibiotics-treated mice were lower than those in normal mice (LCA: 3.5 ± 0.8 vs 1.2 ± 0.7 μg/g, *P* < 0.05) (Fig. 7). In addition, levels of deoxycholic acid (DCA) showed decreasing trend in antibiotics-treated mice (DCA: 47.8 ± 22.6 vs 25.2 ± 7.9 μg/g, *P* = 0.38) (Fig. 7).

## Discussion

The interactions between the composition of the microbiota and gut motility are bidirectional, which indicates that the microbiota play an important role in gut motor function and the gut motor function affects the diversity of microflora in the gastrointestinal tract [6]. Here, we found that the depletion of microbiota by antibiotics treatments was associated with gut motility, and its relationship might be related to 5-HT biosynthesis in the colon. And we also found that metabolism of bile acids was affected in antibiotic-treated mice, and the secondary bile acids was decreased in our study which has been demonstrated to be associated with 5-HT metabolism and gastrointestinal motility [13].

Several studies have focus on the interaction between the normal gut microbiota and gut motility. The dysfunction of gut motility has been considered to be one of the main pathophysiology in STC [31]. Recent years, fecal microbiota transplantation (FMT) has become a new therapy for functional gastrointestinal disorders by engraftment and restoration of normal intestinal microbial community functionality and structure [32]. One prior study showed that FMT had the high efficiency to improve the syndrome of STC [33], which suggested that normal gut microbiota could benefit to gastrointestinal motor function. In addition, Xu et al. [11] also found 5-HT could lead to the contraction of isolated smooth muscle cell, and cell length would be shortened in a 5-HT dose-dependent manner. In our study, after antibiotics treatments, the abundance of gut microbiota decreased significantly in mice, which were also named pseudo-germ-free mice. And antibiotics-treated mice had a decreased intestinal motility, with prolonged gastrointestinal and colonic transit, and poor contraction of smooth muscle in proximal colon. In antibiotics-treated mice, the 5-HT levels in colon was lower, which was consistent with that in a previous study [12]. Thus our findings revealed decreased intestinal motility of mice treated with antibiotics might result from the decreased levels of colonic 5-HT.

The expression of TPH1 in ECs is essential for 5-HT metabolism. Several studies demonstrated that the expression of TPH1 was decreased, but no difference in expression of other steps in 5-HT metabolism was found, like enzymes for 5-HT release [12]. After antibiotics treatments, our results suggested that mice had lower expression of TPH1, and gut microbiota might influence expression of TPH1, which resulted in the decreased level of 5-HT in colon. In addition, we found that there was no difference in the colonic CgA^+^ ECs in both SPF and antibiotics-treated mice, which suggested that the decreased release of 5-HT in “microbiota depletion” by antibiotics was not due to quantity of ECs. Moreover, metabolism modulated by microbiota could change 5-HT biosynthesis in colon, like α-tocopherol, butyrate, cholate, deoxycholate, p-aminobenzoate, propionate, and tyramine [12]. Alemi et al. [13] found that secondary bile acids, like DCA and LCA, caused oral contraction and caudal relaxation, and BAs also led to the release of 5-HT. Interestingly, our study found that secondary bile acids were reduced in antibiotics-treated mice. Thus we proposed that secondary bile acids might influence the expression of TPH1 and then affected the release of 5-HT in colon, which involved in the interaction between microbiota and motility. Further studies are needed to prove whether gut motility would recover after increasing secondary bile acids in mice treated with antibiotics treatments. In addition, gut motility is regulated by multiple signaling pathways with antibiotics, further work is necessary to clarify whether other pathways participate in the regulation of gut motility.

Gradually, studies have started to focus on the relationship between stool consistency and gut microbiota richness [34, 35]. For example, stool consistency was suggested to be negatively correlated with species richness of gut microbiota in humans, which indicated that the fecal parameter, such as water content, was affected not only by gut motility, but also by gut microbiota richness [34]. In our study, the pellet frequency was lower in antibiotics-treated mice, which was consistent with the decreased gut motility. However, the water content was not consistent with the decreased gut motility from the traditional perspective. Although decreased gut motility does increase water reabsorbed from feces in the colon, our study suggested that antibiotics resulted in not only decreased gut motility but also gut microbiota richness which might influence intestinal fluid secretion [36, 37]. Barrett et al. [36] suggested that commensal microbiota exerted important influences on the gastrointestinal epithelial function, such as transporting fluid and electrolytes in the intestine. Further study is needed to explore whether intestinal fluid secretion is affected in antibiotics-treated mice.

## Conclusions

In conclusion, our results from antibiotics-treated mice might differ from some others’, and these might be due to different programs of antibiotics administration, different strains of mice, and environmental conditions. Our findings demonstrated that gut microbiota might play a role in the regulation of secondary BAs and 5-HT metabolism in the colon, which in turn might affect gut motility. These mechanisms may throw new light on the treatment of FMT for functional gastrointestinal disorders.

## Abbreviations

GALT: gut-associated lymphoid tissue
5-HT: 5-hydroxytryptamine
ECs: enterochromaffin cells
TPH: tryptophan hydroxylase
CGRP: calcitonin gene-related peptide
DCA: deoxycholic acid
LCA: lithocholic acid
BAs: bile acids
BSS: Bristol Stool Form Scale
STC: slow transit constipation
FMT: fecal microbiota transplantation

## References

[1] Savage DC (1977). Microbial ecology of the gastrointestinal tract. Annu Rev Microbiol.

[2] Sommer F, Backhed F (2013). The gut microbiota–masters of host development and physiology. Nat Rev Microbiol.

[3] Yang J, Tan Q, Fu Q, Zhou Y, Hu Y, Tang S (2016). Gastrointestinal microbiome and breast cancer: correlations, mechanisms and potential clinical implications. Breast Cancer.

[4] Zhu L, Liu W, Alkhouri R, Baker RD, Bard JE, Quigley EM (2014). Structural changes in the gut microbiome of constipated patients. Physiol Genomics.

[5] Quigley EM (2011). The enteric microbiota in the pathogenesis and management of constipation. Best Pract Res Clin Gastroenterol.

[6] Barbara G, Stanghellini V, Brandi G, Cremon C, Di Nardo G, De Giorgio R (2005). Interactions between commensal bacteria and gut sensorimotor function in health and disease. Am J Gastroenterol.

[7] Barbara G, Feinle-Bisset C, Ghoshal UC, Quigley EM, Santos J, Vanner S (2016). The intestinal microenvironment and functional gastrointestinal disorders. Gastroenterology.

[8] Gershon MD (2013). 5-Hydroxytryptamine (serotonin) in the gastrointestinal tract. Curr Opin Endocrinol Diabetes Obes.

[9] Heredia DJ, Dickson EJ, Bayguinov PO, Hennig GW, Smith TK (2009). Localized release of serotonin (5-hydroxytryptamine) by a fecal pellet regulates migrating motor complexes in murine colon. Gastroenterology.

[10] Fukumoto S, Tatewaki M, Yamada T, Fujimiya M, Mantyh C, Voss M (2003). Short-chain fatty acids stimulate colonic transit via intraluminal 5-HT release in rats. Am J Physiol Regul Integr Comp Physiol.

[11] Xu L, Yu BP, Chen JG, Luo HS (2007). Mechanisms mediating serotonin-induced contraction of colonic myocytes. Clin Exp Pharmacol Physiol.

[12] Yano JM, Yu K, Donaldson GP, Shastri GG, Ann P, Ma L (2015). Indigenous bacteria from the gut microbiota regulate host serotonin biosynthesis. Cell.

[13] Alemi F, Poole DP, Chiu J, Schoonjans K, Cattaruzza F, Grider JR (2013). The receptor TGR5 mediates the prokinetic actions of intestinal bile acids and is required for normal defecation in mice. Gastroenterology.

[14] Ghaffari K, Savadkuhi ST, Honar H, Riazi K, Shafaroodi H, Moezi L (2004). Obstructive cholestasis alters intestinal transit in mice: role of opioid system. Life Sci.

[15] Kawamata Y, Fujii R, Hosoya M, Harada M, Yoshida H, Miwa M (2003). A G protein-coupled receptor responsive to bile acids. J Biol Chem.

[16] Mayer EA, Tillisch K, Gupta A (2015). Gut/brain axis and the microbiota. J Clin Invest.

[17] Hintze KJ, Cox JE, Rompato G, Benninghoff AD, Ward RE, Broadbent J (2014). Broad scope method for creating humanized animal models for animal health and disease research through antibiotic treatment and human fecal transfer. Gut Microbes.

[18] Seki E, De Minicis S, Osterreicher CH, Kluwe J, Osawa Y, Brenner DA (2007). TLR4 enhances TGF-beta signaling and hepatic fibrosis. Nat Med.

[19] Clarke TB, Davis KM, Lysenko ES, Zhou AY, Yu Y, Weiser JN (2010). Recognition of peptidoglycan from the microbiota by Nod1 enhances systemic innate immunity. Nat Med.

[20] Baldridge MT, Nice TJ, McCune BT, Yokoyama CC, Kambal A, Wheadon M (2015). Commensal microbes and interferon-lambda determine persistence of enteric murine norovirus infection. Science.

[21] Arumugam M, Raes J, Pelletier E, Le Paslier D, Yamada T, Mende DR (2011). Enterotypes of the human gut microbiome. Nature.

[22] Xu H, Hao W, Zhou Q, Wang W, Xia Z, Liu C (2014). Plaque bacterial microbiome diversity in children younger than 30 months with or without caries prior to eruption of second primary molars. PLoS ONE.

[23] Schloss PD, Westcott SL, Ryabin T, Hall JR, Hartmann M, Hollister EB (2009). Introducing mothur: open-source, platform-independent, community-supported software for describing and comparing microbial communities. Appl Environ Microbiol.

[24] Li Z, Chalazonitis A, Huang YY, Mann JJ, Margolis KG, Yang QM (2011). Essential roles of enteric neuronal serotonin in gastrointestinal motility and the development/survival of enteric dopaminergic neurons. J Neurosci.

[25] Nezami BG, Mwangi SM, Lee JE, Jeppsson S, Anitha M, Yarandi SS (2014). MicroRNA 375 mediates palmitate-induced enteric neuronal damage and high-fat diet-induced delayed intestinal transit in mice. Gastroenterology.

[26] He WQ, Peng YJ, Zhang WC, Lv N, Tang J, Chen C (2008). Myosin light chain kinase is central to smooth muscle contraction and required for gastrointestinal motility in mice. Gastroenterology.

[27] He WQ, Qiao YN, Peng YJ, Zha JM, Zhang CH, Chen C (2013). Altered contractile phenotypes of intestinal smooth muscle in mice deficient in myosin phosphatase target subunit 1. Gastroenterology.

[28] Ichinohe T, Pang IK, Kumamoto Y, Peaper DR, Ho JH, Murray TS (2011). Microbiota regulates immune defense against respiratory tract influenza A virus infection. Proc Natl Acad Sci USA.

[29] Rakoff-Nahoum S, Paglino J, Eslami-Varzaneh F, Edberg S, Medzhitov R (2004). Recognition of commensal microflora by toll-like receptors is required for intestinal homeostasis. Cell.

[30] Chen JJ, Li Z, Pan H, Murphy DL, Tamir H, Koepsell H (2001). Maintenance of serotonin in the intestinal mucosa and ganglia of mice that lack the high-affinity serotonin transporter: abnormal intestinal motility and the expression of cation transporters. J Neurosci.

[31] Rao SS, Rattanakovit K, Patcharatrakul T (2016). Diagnosis and management of chronic constipation in adults. Nat Rev Gastroenterol Hepatol.

[32] Khoruts A, Sadowsky MJ (2016). Understanding the mechanisms of faecal microbiota transplantation. Nat Rev Gastroenterol Hepatol.

[33] Tian H, Ding C, Gong J, Ge X, McFarland LV, Gu L (2016). Treatment of slow transit constipation with fecal microbiota transplantation: a pilot study. J Clin Gastroenterol.

[34] Vandeputte D, Falony G, Vieira-Silva S, Tito RY, Joossens M, Raes J (2016). Stool consistency is strongly associated with gut microbiota richness and composition, enterotypes and bacterial growth rates. Gut.

[35] Hadizadeh F, Walter S, Belheouane M, Bonfiglio F, Heinsen FA, Andreasson A (2016). Stool frequency is associated with gut microbiota composition. Gut.

[36] Barrett KE (2016). Endogenous and exogenous control of gastrointestinal epithelial function: building on the legacy of Bayliss and Starling. J Physiol.

[37] Lundgren O (1998). 5-Hydroxytryptamine, enterotoxins, and intestinal fluid secretion. Gastroenterology.

